# Supplementary material for: Are carbon emissions trading and green financial instruments synergistic? -Comprehensive quantitative research based on content analysis
Source: PLoS One. 2024 Mar 7;19(3):e0298601. doi: 10.1371/journal.pone.0298601 (PMC10919601; doi:10.1371/journal.pone.0298601)
Supplement: S1 Data — (ZIP) [file pone.0298601.s001.zip › Data/地级市Z_F工作报告.zip!/╔╜╢1⁄2/╤╠╠¿╩╨/└┤╫╘╣1⁄2╓┌║┼í╛┬φ┐╦╩2╛▌═°í┐_2014.docx]

各位代表：

现在，我代表市人民政府向大会报告政府工作，请予审议，并请市政协委员和其他列席会议的同志提出意见。

一、2013年工作回顾

过去的一年，在中共烟台市委的正确领导下，我们认真贯彻落实国家、省一系列改革创新举措，出台实施一批综合性和专项性政策措施，努力推动经济社会发展实现稳中有进、稳中向好，各项工作取得新的成绩。预计，全市生产总值5613亿元，按可比价格计算比上年增长10.2%;公共财政预算收入437.2亿元，可比口径增长12.6%;城市居民人均可支配收入和农民人均纯收入分别达到32930元和14761元，分别增长9.6%和11%;居民消费价格涨幅控制在1.8%。主要做了以下工作：

(一)强化稳增长措施，经济实现平稳较快发展。面对复杂的经济形势，更加注重扩消费、促投资、稳外需的协同发力，更加注重激发区域发展活力。积极培育消费增长点，加快推进30个重大商贸项目，新建一批便民商贸设施，社会消费品零售总额2103亿元，增长13.1%。大力发展电子商务，年交易额增长34%。着力促进投资合理增长和结构优化，加快建设200个市级重点项目，青烟威荣城际铁路、龙烟铁路、新机场、烟台港西港区、海阳核电等重点工程顺利推进，潍莱高速铁路列入全省快速铁路建设规划，全社会固定资产投资3525亿元，增长20.1%。千方百计扭转外贸下滑局面，一般贸易出口增长15%，高新技术产品出口增长35%，进出口总额493亿美元，增长3.1%。实际使用外资16亿美元，增长13.5%。现代汽车研发中心是目前全省最大的外资研发机构，台湾鸿海集团10个新合作项目陆续投产。境外投资3.6亿美元，增长33.3%。新缔结2个国际友好城市。全力推动区域经济崛起，东部新区加快建设，“海洋经济发展中心”主体封顶，16横21纵路网工程全线推进，产业招商初见成效。莱州湾、丁字湾、龙口湾基础设施和产业项目快速建设，长岛获批国家级海洋生态文明示范区和国家级海洋公园。我市在全省蓝色经济区绩效考核中获第二名，莱州市在全省黄河三角洲高效生态经济区绩效考核中获第一名。烟台开发区连续第6年进入国家级开发区综合评价前六强，高新区进入科技部创新型特色园区创建试点，保税港区主要经济指标增幅高于全国保税港区平均水平，招远开发区加快转型、提质发展，7个省级开发区实现进位发展，其中龙口开发区居省级开发区排名首位。促进县域经济创新跨越，扩权强镇改革取得新进展。

(二)加快“转调创”步伐，发展质量效益持续提升。推动工业转型升级，万华、张裕工业园等一批重点项目快速建设，800多个企业完成新一轮技术改造。5个传统优势产业、5个战略性新兴产业主营业务收入分别增长12%和20%，规模以上工业主营业务收入突破1.4万亿元，利税、利润分别增长12%和11.8%，形成了5个千亿规模产业、3个千亿规模产品集群、1个千亿规模企业、16个百亿规模企业。加快提升现代服务业，大力推进50个服务业重点项目。全年接待游客5043万人次，第四次荣获中国最佳休闲城市称号。成功举办各类会展142个，第十四届国际果蔬食品博览会实行市场化运作，荣获全国优秀会展城市奖。新增金融机构5家、小额贷款公司3家、创投企业5家。本外币存款余额6021亿元，增长13.8%。6家企业分别在A股市场过会或区域股权交易市场挂牌，上市公司再融资21.5亿元。完成港口货物吞吐量2.86亿吨、集装箱吞吐量215万标箱。服务业增加值占生产总值比重提高1.4个百分点。创新发展现代农业，发放涉农补贴3.6亿元，农业生产再获丰收。蓬莱农业高新技术产业示范区晋升为国家级农业科技园区。完成农村集体土地确权颁证工作。全社会水利投入创历史最高水平。大力推动科技创新，新增高新技术企业45家、省级以上工程技术研究中心和企业技术中心35家，高新技术产业产值在工业总产值中占比达到40%。新增1件国家地理标志证明商标、34件著名商标、43个山东名牌和山东服务名牌。引进各类人才3万人，聘请11名政府科技顾问。获得国家科技进步二等奖3项、国家技术发明二等奖1项、省科学技术奖43项、中国专利优秀奖5项。第五次荣获全国科技进步先进市。启动“财税质量三年提升计划”，税收占公共财政预算收入比重提高3.7个百分点。

(三)实施重点领域改革，发展活力不断增强。认真学习贯彻中央、省委关于全面深化改革的《决定》、《意见》，结合实际研究制定我市改革意见。开展第六轮行政审批制度改革，取消、下放行政审批事项286项，总量减少40.5%。深化国有企业改革，建立国有资本经营预算制度，市管企业资产突破1200亿元，增长15%。鼓励投资创业，新登记各类市场主体增长19.3%。开展户籍管理制度改革，实行居住登记和居住证制度。深化医药卫生体制和医疗服务价格改革，5家县级公立医院综合改革试点工作顺利推进，基本药物制度覆盖80%以上的村卫生室。实施食品药品工商质监和金融管理体制改革。

(四)推进新型城镇化，城乡统筹步伐明显加快。出台实施城镇化发展纲要，探索具有胶东特色的功能区带动型城乡一体化发展模式。编制各类发展规划110多项。524项城建重点工程启动建设，开展48个区片和233个城中(郊)村改造，实施一批城乡道路、停车场、公交场站和过街天桥建设改造工程。中心城区实现道路交通智能化管理服务全覆盖。启动建设5项污水、垃圾处理工程。完成88项耕地占补平衡和土地综合整治项目。深入开展生态文明示范区和生态文明乡村创建工作，建设农村新型社区156个，完成800个村庄村容村貌综合整治任务，新建改建农村公路1137公里。我市被评为全国绿化模范城市，昆嵛山保护区入选首批国家生态旅游示范区。开展“蓝天白云”行动，实施河道及水源地环境综合治理，推进78项大气和81项水污染减排工程，新投用238辆环保型公交车，市区空气质量优良率超过90%。在经济总量取得突破的同时完成了节能减排任务。在《2013中国绿色发展指数报告》中我市列100座测评城市第五位。

(五)着力保障和改善民生，各项社会事业全面进步。公共财政预算民生支出增长15%。各级政府承诺的为民服务实事全部完成。新增城镇就业17万人，转移农村劳动力3.3万人。企业退休人员基本养老金、城乡居民基础养老金和基本医保财政补助、城乡低保、农村五保和孤儿供养、最低工资、失业金领取标准进一步提高。启动新农合和城镇居民大病保险。为709名残疾儿童进行抢救性康复救助，为4.3万名小学生实施龋病预防干预。完成223项库区村民生工程。发放救助款物2.38亿元，慈善义工发展到3.7万人。11处社会福利中心投入使用。筹建保障房4028套，完成棚户区改造2.58万户。教育事业健康发展，开工建设133所乡镇公办中心幼儿园，义务教育段学校学生免费校服覆盖面达到83%、6个县市区开展了免费午餐试点。新增养老机构24处、床位5600张，新建社区老年人日间照料中心56处，65周岁以上老人享受免费乘坐公交车待遇。新建、改扩建烈士纪念设施10处。成功举办第二届烟台文化艺术节，圆满完成第十届中国艺术节承办任务，4件群众文化作品荣获“群星奖”。获批国家历史文化名城，入选文化强省建设先进市、国家公共文化服务体系示范区创建城市。“四德工程”在全国推广，刁娜入选第四届“全国道德模范”。“全国全民健身示范城市”试点工作通过验收，成功举办烟台市第八届运动会，我市体育健儿在第十二届全运会上荣获24.5枚金牌，创历届全运会之最。国防动员、民兵预备役、人民防空和双拥共建工作有序开展，人口计生、老龄、妇女、儿童、残疾人、红十字事业健康发展，统计、史志、档案、仲裁、新闻出版、广播电视、民族宗教、气象、地震等工作取得新成绩。开展平安烟台、法治烟台、和谐烟台“三城联创”，安全生产和食品药品安全形势持续稳定，信访、综治工作扎实有效，连续第六次荣获全国社会管理综合治理优秀城市，在“2013年度中国最安全城市”评比中列第五位。

各位代表，过去的一年，各级面对新形势、新变化，积极主动采取应对措施，深处着力、精准发力，进一步激发了经济社会发展活力。针对关键领域和薄弱环节，连续出台培育发展市场主体23条、服务业21条、旅游业20条、金融业26条、电子商务27条、健康服务业16条、养老产业18条以及创新园区发展21条和公共服务体系建设42条等政策措施，动员各方面抓产业促发展，分工推进对外开放各项具体工作，进一步推动了市场主体的增量提升、新兴产业的快速发展、对外经贸的优化升级和服务群众工作的改进创新。抓住国内外大企业转型升级的机遇，筛选一批央企、省企、外企、民企以及高等院校和科研院所，集中开展“大走访”活动，跟踪落实香港山东周等重大经贸活动达成的协议项目。面对企业发展遇到的困境，在行政审批、融资担保、鼓励出口、刺激消费、税费改革等方面出台了一系列帮扶政策，集中64个窗口开展“一站式”通关服务，分别安排1.5亿元和1.8亿元资金支持企业技术改造、扶持外经贸提升发展，组织1万多户企业实施“营改增”改革试点，循环发放企业信贷周转金8亿元，对2.6万户小微企业免征增值税和营业税，减轻企业负担40多亿元。围绕加强政府自身建设，严格执行中央、省、市改进作风的各项规定，修订出台政府工作规则等规章制度，扎实开展工作落实年、治理“庸懒散”等活动。厉行节约，反对浪费，市级会议费和“三公”经费下降22.7%。强化行政监察和审计监督，纠正不正之风，惩治腐败行为。认真执行市人大及其常委会各项决议、决定，主动加强与市政协联系，自觉接受社会各界监督，办复市人大代表建议86件、政协提案599件。有效组织了“我为《政府工作报告》建言献策暨2014年‘为民服务实事’征集活动”，广泛听取民意、了解民愿、集中民智，增强了政府工作的针对性和实效性。

各位代表，过去一年的成绩来之不易。这是党中央国务院、省委省政府和市委科学决策、正确领导的结果，是市人大、政协和社会各界有效监督、支持参与的结果，是广大干部职工和全市人民真抓实干、共同努力的结果。在此，我代表市人民政府，向全市人民，向市人大代表、政协委员，向各民主党派、工商联、各人民团体和社会各界人士，向中央省属驻烟单位、驻烟人民解放军和武警部队官兵，向老领导、老同志，向所有关心支持烟台发展的海内外朋友们，表示衷心的感谢和崇高的敬意!

当前，我市经济社会发展还面临不少困难和挑战，政府工作依然存在一些差距和不足。经济稳定发展的基础还不牢固，外需仍然不足，内需增长乏力;产业结构调整升级任务艰巨，服务业快速发展需要付出很大努力;资金配置不尽合理，融资渠道还不够多;市场主体成长不够快、活力不够足;经济增长与资源环境约束之间的矛盾日益显现，环境治理力度需要进一步加大;城乡及区域之间发展不平衡问题仍然突出;政府公共服务能力和社会治理水平与人民群众的期望相比还有差距;少数工作人员作风漂浮，等等。这些问题都必须尽快加以解决。

二、2014年政府工作总体思路和目标任务

今年改革与发展的任务重大而艰巨。政府工作的总体思路是：以邓小平理论、“三个代表”重要思想、科学发展观为指导，深入贯彻落实党的十八大和十八届二中、三中全会精神，按照习近平总书记系列重要讲话和视察山东重要讲话要求，紧紧围绕“一个率先、三个坐标、三个跨越”的奋斗目标，以提高经济发展质量和效益为中心，坚持稳中求进、改革创新，积极推动工作指导重大转变，坚决打好转方式调结构攻坚战，实现改革开放新突破，构建现代产业新体系，增创区域发展新优势，激发市场主体新活力，开创城乡建设新局面，促进社会建设新进步，推动经济持续健康发展、社会和谐稳定。

今年经济社会发展的主要预期目标是：全市生产总值增长9.5%左右;公共财政预算收入增长11.5%左右;全社会固定资产投资增长18%;社会消费品零售总额增长13%;实际使用外资增长10%，进出口增长6%左右;城市居民人均可支配收入和农民人均纯收入均增长10%左右;城镇登记失业率控制在3.5%以内;居民消费价格涨幅控制在3.5%左右;人口自然增长率控制在2.4‰以内;完成省下达的年度节能减排任务。

实际工作中，我们将按照习近平总书记对山东提出的“努力在推动科学发展、全面建成小康社会进程中走到前列”，“努力在转变经济发展方式、全面提高经济发展质量和效益上起到领头雁作用”的新要求，牢牢把握“稳中求进、改革创新”的基本原则，切实转变观念，努力实现质量和效益切实得到提高的经济发展速度。

各位代表，新的一年，是烟台经济社会发展史上极为重要的一年。今年是全面深化改革的开局之年，我们将按照党的十八届三中全会，省委十届七次、八次全会，市委十二届四次全会的战略部署，坚定不移推进新一轮改革，把改革创新贯穿于经济社会发展各个领域各个环节，充分发挥改革对经济社会的牵引作用。今年是我市获批全国首批沿海开放城市三十周年，我们将依托三十年来积聚的开放经验、产业基础、资源优势和人才队伍，以国际的标准提升工作，以世界的眼光谋划未来，努力扩大招商领域、提高合作层次、完善开放体制，在新的历史起点上开创对外开放新局面。今年是我市重大基础设施实现历史性突破的一年，经过几届党委政府和全市上下的长期不懈努力，年内青烟威荣城际铁路、新机场将会竣工，烟台港西港区至淄博原油管道全线贯通，龙烟铁路开工建设控制性工程，海阳核电1、2号机组进入设备安装调试高峰阶段，南水北调配套工程全线推进，烟台发展的交通环境和支撑条件将实现历史性突破。我们坚信，只要全市上下团结一心、众志成城，形成共促改革、共谋发展、共创和谐的强大合力，就一定能够创造科学发展的新辉煌!

三、2014年重点工作

(一)全面深化改革扩大开放。改革开放成就了烟台的昨天，影响着烟台的今天，决定着烟台的明天。抓住全面深化改革的重大机遇，以改革开放激发增长动力和发展活力。

深入贯彻落实中央、省、市全面深化改革的《决定》、《意见》，正确、准确、有序、协调推进各领域改革，到2020年在重要领域和关键环节改革上取得决定性成果，力争较多领域走在全省乃至全国改革前列。围绕使市场在资源配置中起决定性作用深化经济体制改革，完善国有资产管理体制，健全促进民营经济发展机制，积极发展混合所有制经济。实行统一市场准入制度，改革注册资本登记制度，激发和释放市场主体活力。完善主要由市场决定价格机制，加快公用公益事业价格改革。围绕转变政府职能，推进行政审批制度改革，强化公共服务，优化政府组织机构。深化财税体制改革，稳步提高财政收入质量，加强预算支出管理。围绕推进城乡发展一体化，统筹城乡综合配套改革，创新现代农业经营体制和农村产权制度，促进城乡要素平等交换，深化户籍制度和小城镇管理体制改革，完善城镇化健康发展机制和区域协调发展机制，推进土地管理制度改革，建立城乡统一的建设用地市场。围绕推进社会事业创新发展，进一步深化科技、教育、文化、卫生、就业、分配、社会保障等各领域改革。

以开放促改革促发展是改革开放以来的成功经验。适应经济全球化新形势，树立“大开放”理念，着力推进“五个国际化”，加快构建开放型经济新体制。大力推进产业国际化。着眼国际市场进行产业定位、产业招商和产业链延伸，建立更具国际竞争优势的产业体系。继续深入开展“大走访”活动，强化与海内外大企业、高等院校和科研院所的战略合作，推动在谈项目及早落地。积极引进外资参与产业转型升级，拓展利用外资新领域，推动服务业有序开放，提高工业、农业利用外资水平。大力推进市场国际化。深入实施以质取胜和市场多元化战略，推行价值链升级导向的对外贸易。进一步优化出口结构，引导企业更多参与国际市场竞争，支持企业以新技术、新产品开拓国际市场。扩大资源能源、先进材料及技术设备和高品质消费品进口。完善“大通关”综合服务。积极发展服务贸易，争创跨境贸易电子商务服务试点城市和国家服务外包示范城市。大力推进企业国际化。支持有条件的企业到境外上市或发行债券。支持有实力的企业全球布局产业链，开展绿地投资、并购投资，实施国际研发合作，建立全球营销网络，打造源自烟台的跨国公司和国际品牌。支持企业以外贸的理念和标准发展内贸，实现内外贸一体化发展。大力推进园区国际化。积极引导境内外资本参与园区建设，支持园区进行跨国合作，开展省级以上园区体制机制创新试点，探索建设行政审批“零收费”园区，建立“飞地”园区机制，促进园区进位升级。鼓励烟台开发区在深化改革、扩大开放中走在全省全国开发区前列，支持高新区打造科技引擎和创新高地，推动保税港区争取自由贸易等试点政策，促进招远开发区创建国家生态工业示范园区，激励各类园区健康快速发展。大力推进城市国际化。扩大对外交流与合作，开展招商推介活动搭建开放平台，抓住担任东亚经济推进机构主席城市机遇加深区域交流，进一步拓展与日韩两国、港台两地、欧美两洲及其他重点区域的合作深度和广度。增辟国际客货运航线，推进中韩铁路轮渡项目，促进中日韩地方经济合作示范区建设。积极稳妥推进金融业对外开放。大力推动中国农业大学与爱尔兰都柏林大学在我市合作办学。规划建设国际化居住社区、教育医疗机构和城市综合服务体系，营造稳定、透明、公平和舒适方便的外商投资及生活环境。

(二)加快推进产业结构调整。经济转型升级是事关烟台前途命运的一场硬仗。按照高端高质高效的方向，积极推动三次产业结构由“二三一”向“三二一”转变，加快构建富有烟台特色、充满创新活力、具有核心竞争力的现代产业体系。

我市农业特色鲜明，必须探索体现地域优势的农业现代化道路。巩固农业基础地位，使之与工业化、城镇化和信息化的发展相协调。坚持以“高产、优质、高效、生态、安全”为目标，扩大水果、水产、蔬菜等产业优势，推进“粮安工程”和“放心粮油工程”，加强动物无规定疫病区建设，健全肉菜流通追溯体系，保护和提升烟台苹果、烟台海参、龙口粉丝、莱阳梨等传统优势品牌，高标准打造精品葡萄酒主产区，大力发展乡村旅游和休闲农业。推动农业科技进步和创新，促进种子种苗产业规模化发展。培育家庭农场、专业大户、农民合作社、龙头企业等新型经营主体，开展职业农民培训，发展农业社会化服务体系。加强农业标准化生产，将农产品质量安全纳入对县乡政府考核，用严谨的标准、严格的监督、严厉的处罚、严肃的问责，维护我市中国食品名城、绿色食品城的美誉。提升农机装备水平。扩大政策性农业保险实施范围和规模。稳定土地承包关系，加快农村土地承包经营权确权登记颁证工作，开展集体土地范围内房屋登记。实施10座大中型水库除险加固工程，发展节水灌溉35万亩，建设旱涝保收高标准农田20万亩。

工业是我市的支柱产业，创新发展的方向是做优做精传统产业、做大做强新兴产业。充分发挥企业主体和市场主导作用，坚持以“产业链延伸、价值链提升、供应链优化”为导向，深入实施“5510工程”、“8515工程”，推动机械、电子、食品、黄金、化工等传统优势制造业向精细工业转型，引导企业向以加工制造为基础的综合服务商转型。支持行业领军企业做大做强，推进75个投资过3亿元的重点工业项目和100个投资过亿元的技改项目建设，加快工业企业“退城进园”步伐，实现万华、张裕工业园新建生产线部分投产。化解工业产能过剩，探索建立远近统筹、标本兼治的产能进退衔接长效机制。推动信息化与工业化深度融合，组建物联网企业联盟，抓好一批重点企业和县市区“两化融合”试点。大力发展新兴制造业，扶持新材料、高端装备制造、生物技术、节能环保等产业快速发展，实施杰瑞页岩气压裂装备产业化等一批新兴产业项目，打造聚氨酯基地、新型铝材产业示范基地、生物科技产业基地、海洋工程装备基地、汽车及零部件基地和低碳产业基地。

服务业是经济转型升级的突破口，也必将成为未来经济发展的新引擎。坚持以“发展提速、增长提质、比重提高、效益提升、水平提档”为目标，吸引各类社会资本平等进入服务业领域，重点培育高端服务业，突出抓好30个服务业重点园区、20条特色街、100个重点企业和200个重点项目。大力发展更具竞争优势的商贸流通业。繁荣城市商圈和中央商务区，加快形成以总部经济、传媒创意、商务服务为主题的楼宇群，推进建设10个大型商贸设施，新引进10个国际著名品牌。采取市场化模式办好会展活动。支持住宿和餐饮业健康发展。鼓励机关及企事业单位后勤服务社会化。大力发展具有烟台特色的旅游业。抓住交通改善机遇，着力开发旅游产品，建设一批旅游项目，开展“智慧旅游年”活动，加强海内外市场营销，全面打造“山海仙境·葡萄酒城”的旅游品牌。大力发展体现区位优势和陆海空交通优势的现代物流业。重点规划建设一批物流园区，逐步完善覆盖全市的便民物流网络。大力发展具有烟台特色的现代金融业。坚持以“做全门类、做强机构、做大规模、做好服务”为目标，加强金融生态建设，健全地方金融监管体制，积极稳妥推进金融创新，促进金融业健康快速发展。加快地方法人金融机构综合改革和转型发展，充分放大本地金融机构总部优势，积极推动设立和引进各类金融机构，有序推进民间融资阳光化试点。筹备设立海洋资产交易平台、农村产权流转交易平台、金融服务外包平台，探索发展介于现货与期货之间的大宗商品交易市场，新设一批产业投资基金。加大债务融资力度，扩大债券发行规模。积极推动企业上市，支持已上市企业并购重组和集团整体上市，着力打造A股市场、新三板市场以及香港等境外市场上令人瞩目的烟台板块。大力发展以信息技术创新为支撑的信息消费业和电子商务产业。加快建设烟台电子商务产业园，创建一批电子商务平台，引进发展一批电子商务龙头企业，鼓励企业和群众开设更多的网上商店。建立健全“12343”等民生服务体系。大力发展具有地域特色和时代特色的健康服务业、养老产业。鼓励社会资本兴办医疗健康和养老机构，打造全国一流的医疗健康产业基地式城市和养老产业典范城市。规划发展科研、文化、商务、教育、体育、对外贸易、人力资源、社区服务等各种行业的服务经济。构建扩大服务性消费的长效机制，提高消费支出在国民经济中的比重。
